# Predator–prey mass ratio drives microbial activity under dry conditions in *Sphagnum* peatlands

**DOI:** 10.1002/ece3.4114

**Published:** 2018-05-12

**Authors:** Monika K. Reczuga, Mariusz Lamentowicz, Matthieu Mulot, Edward A. D. Mitchell, Alexandre Buttler, Bogdan Chojnicki, Michał Słowiński, Philippe Binet, Geneviève Chiapusio, Daniel Gilbert, Sandra Słowińska, Vincent E. J. Jassey

**Affiliations:** ^1^ Laboratory of Wetland Ecology and Monitoring Faculty of Geographical and Geological Sciences Adam Mickiewicz University Poznań Poland; ^2^ Department of Biogeography and Palaeoecology Faculty of Geographical and Geological Sciences Adam Mickiewicz University Poznań Poland; ^3^ Faculty of Biology Adam Mickiewicz University Poznań Poland; ^4^ Laboratory of Soil Biodiversity University of Neuchatel Neuchatel Switzerland; ^5^ Jardin Botanique de Neuchâtel Neuchatel Switzerland; ^6^ Swiss Federal Research Institute WSL Site Lausanne Lausanne Switzerland; ^7^ Laboratoire des Systèmes Écologiques School of Architecture, Civil and Environmental Engineering École Polytechnique Fédérale de Lausanne (EPFL) Lausanne Switzerland; ^8^ Laboratoire de Chrono‐Environnement UMR CNRS 6249 UFR des Sciences et Techniques Université de Franche‐Comté Besançon France; ^9^ Department of Meteorology Faculty of Environmental Engineering and Spatial Management Poznan University of Life Sciences 60‐649 Poznań Poland; ^10^ Department of Environmental Resources and Geohazard Polish Academy of Sciences Institute of Geography and Spatial Organization Warszawa Poland; ^11^ Department of Geoecology and Climatology Polish Academy of Sciences Institute of Geography and Spatial Organization Warsaw Poland; ^12^ Laboratoire d'Ecologie Fonctionnelle et Environnement (Ecolab) INPT, UPS, CNRS Université de Toulouse Toulouse Cedex France; ^13^ UMR CARRTEL INRA 042 University of Savoie Mont‐Blanc FR‐ 73376 Le Bourget du lac France

**Keywords:** body size, drought, food web, phenoloxidase, poor fen, protists, soil moisture, water table manipulation, Wetlands

## Abstract

Mid‐ to high‐latitude peatlands are a major terrestrial carbon stock but become carbon sources during droughts, which are increasingly frequent as a result of climate warming. A critical question within this context is the sensitivity to drought of peatland microbial food webs. Microbiota drive key ecological and biogeochemical processes, but their response to drought is likely to impact these processes. Peatland food webs have, however, been little studied, especially the response of microbial predators. We studied the response of microbial predators (testate amoebae, ciliates, rotifers, and nematodes) living in *Sphagnum* moss carpet to droughts, and their influence on lower trophic levels and on related microbial enzyme activity. We assessed the impact of reduced water availability on microbial predators in two peatlands using experimental (Linje mire, Poland) and natural (Forbonnet mire, France) water level gradients, reflecting a sudden change in moisture regime (Linje), and a typically drier environment (Forbonnet). The sensitivity of different microbial groups to drought was size dependent; large sized microbiota such as testate amoebae declined most under dry conditions (−41% in Forbonnet and −80% in Linje). These shifts caused a decrease in the predator–prey mass ratio (PPMR). We related microbial enzymatic activity to PPMR; we found that a decrease in PPMR can have divergent effects on microbial enzymatic activity. In a community adapted to drier conditions, decreasing PPMR stimulated microbial enzyme activity, while in extreme drought experiment, it reduced microbial activity. These results suggest that microbial enzymatic activity resulting from food web structure is optimal only within a certain range of PPMR, and that different trophic mechanisms are involved in the response of peatlands to droughts. Our findings confirm the importance of large microbial consumers living at the surface of peatlands on the functioning of peatlands, and illustrate their value as early warning indicators of change.

## 1 INTRODUCTION

Over the past few decades, climate warming has caused numerous hydroclimate anomalies in northern latitudes resulting in dramatic hydrological shifts at regional and local scales (Ljungqvist et al., 2016). Model simulations predict further increases in the intensity and frequency of droughts along with warmer temperatures (Dai, 2013). Such changes may have devastating implications for northern ecosystems, many of which are especially dependent on water balance (Turetsky et al., 2017). Northern peatlands are particularly vulnerable to droughts as their water balance is mostly controlled by precipitation, and this vulnerability may have global consequences (Dise, 2009). Northern peatlands store vast amounts of terrestrial carbon (C) (*ca*. 450 Gt C) as peat (Frolking & Roulet, 2007; Gorham, 1991; Yu, 2012). The moist and acidic conditions prevailing in these ecosystems slow down microbial activities, thus leading to an imbalance between primary production and decomposition rates (Frolking et al., 2001). However, more frequent and intense droughts are likely to profoundly and durably alter peatland C balance due to the response of microbial communities. Drought may accelerate the rate of organic matter decomposition (Fenner & Freeman, 2011), thereby releasing previously locked C and turning peatlands from C sinks into C sources (Davidson & Janssens, 2006).

Understanding the response of peatland soil microbial communities to drought is of major importance because of their key role as decomposers. Drought often enhances the activity of peatland microorganisms (Fenner & Freeman, 2011), concomitantly with shifts in communities’ composition (Nunes et al., 2015). However, in some peatlands, drought had only little impact on bacterial and fungal communities (Peltoniemi et al., 2012). The duration and intensity of droughts are important factors potentially explaining such differences in response, but effects on higher trophic levels within microbial communities may also be relevant (Trap, Bonkowski, Plassard, Villenave, & Blanchart, 2016). Indeed, in addition to bacteria and fungi, peat‐forming mosses host a high diversity of microbial consumers such as ciliates, testate amoebae, rotifers, and nematodes (Kostka et al., 2016). Fungal and bacterial populations, among other functions, constitute the main food resource for these diverse consumers. Ciliates, rotifers, and nematodes are the main bacterial and fungal feeders (Gilbert, Amblard, Bourdier, & Francez, 1998; Mieczan, 2007), while testate amoebae feed on a larger variety of prey, including bacteria, fungi, protists, rotifers, and nematodes (Gilbert, Mitchell, Amblard, Bourdier, & Francez, 2003; Yeates & Foissner, 1995).

Many of these microbial consumers are adapted to life in permanently waterlogged conditions and are thus vulnerable to hydrological shifts, as shown for testate amoebae in palaeoecological records (Lamentowicz et al., 2015). Drought imposes direct physiological stress on soil microorganisms, leading to the local disappearance of sensitive species (Schimel, Balser, & Wallenstein, 2007). The resulting shifts in species occurrences and abundances are likely to generate community re‐assembly (Bardgett, Manning, Morriën, & De Vries, 2013), novel species interactions, and foraging dynamics (Mason, Brivio, Stephens, Apollonio, & Grignolio, 2017), with unknown but potentially powerful impacts on food web stability (Ledger, Brown, Edwards, Milner, & Woodward, 2013). Along with reduced species abundances or local extinctions, tolerant species may experience resource limitation, forcing them to re‐allocate resources (Schimel et al., 2007), which again, may impact their fitness and feedback to food web architectures and dynamics (Lu et al., 2016). The biomass of species is key to understanding such changes because it incorporates both individual growth rates and interactions among species (e.g., feeding paths; Woodward et al., 2010; Yvon‐Durocher, Montoya, Trimmer, and Woodward, 2011). For example, a loss of species with high standing biomass, such as the top predators, can lead to corresponding trophic cascade prey release and then alter the C dynamics of ecosystems (Staddon, Lindo, Crittenden, Gilbert, & Gonzalez, 2010). This could be mitigated, however, when compensatory mechanisms such as increased production of smaller predators or species gain preserve the predator‐to‐prey mass ratio (Schneider, Scheu, & Brose, 2012), thus maintaining ecosystem functions in the face of disturbance despite a reconfiguration of the food web architecture (Ledger et al., 2013).

The consequences of drought on peatland microbial consumers and their body mass structure remain largely unknown. Most studies focused on low trophic levels (bacteria and fungi) (Jaatinen, Fritze, Laine, & Laiho, 2007; Nunes et al., 2015) and little empirical evidence exist for predicting future changes within multiple trophic levels (Lindo, 2015). Yet, some studies showed the importance of trophic interactions among multiple trophic levels in driving soil decomposition processes (Sauvadet et al., 2016). Here, we address this gap in understanding by examining the response of peatland microbial consumers to drought and the consequences on soil extracellular enzyme activity. In particular, we tested the relationship between microbial enzymatic activity and the predator‐to‐prey mass ratio (PPMR) as it captures how shifts in the feeding links within the food web might influence the activity of basal prey (bacteria and fungi). For example, a decrease in PPMR might suggest an increase in basal prey biomass (e.g., decomposers), which in turn, would stimulate microbial activity.

We hypothesize that (1) the biomass of larger microbial consumers will decrease along with water level drop, thus decreasing PPMR value. (2) We expect such a decrease in larger microbial consumers to promote the biomass production of smaller microbial consumers (bacterial and fungal feeders) due to top‐down prey release effects. Basal prey populations (bacteria and fungi) may, however, decrease if drought impacts preferentially the larger microbial predators which feed primarily on protists and micro‐metazoa as opposed to bacteria and fungi (Jassey, Shimano, Dupuy, Toussaint, & Gilbert, 2012). We further expect (3) that PPMR will be negatively related to extracellular enzyme activity. This will indicate that a decrease in microbial consumers stimulates bacterial and fungal activity due to a lower predation pressure from bacterivores and fungivores. Furthermore, peat mosses are characterized by vertical gradients of moisture and microbial communities’ structure along the first few centimeters (Meisterfeld, 1977; Mitchell & Gilbert, 2004). With respect to the vertical micro‐gradient, we expected (4) that the effects of drought on microbial consumers and related relationships with decomposers and enzymes would be less pronounced with depth. We tested the above hypotheses using an observational study along a hydrological gradient and an experimental field study where different moisture conditions were tested. We focused on phenoloxidases, which are among the few enzymes able to degrade recalcitrant material such as phenolic compounds (McLatchey & Reddy, 1998), and as such play a key role in peatland decomposition processes (Freeman, Ostle, & Kang, 2001).

## 2 MATERIALS AND METHODS

### 2.1 Study sites and drought simulation

The research was conducted in two *Sphagnum‐*dominated peatlands: Forbonnet (Jura Mountains, north‐eastern France, 46°49′35″N, 6°10′20″E) and Linje (Complex of Chełmno and Vistula Landscape Parks, northern Poland, 53°11′15″N, 18°18′34″E) (Figure S1). The overall plant species composition and hydrochemical conditions were comparable between these two sites (Buttler et al., 2015; Lamentowicz et al., 2016). The two sites also showed similar pH (see Table 1). To study th effect of drought, we used two different designs: a natural moisture gradient in Forbonnet (Bragazza et al., 2016), where microbial communities adapted to naturally dry conditions are compared to microbial communities adapted to wet conditions, and an abrupt variation in water table in Linje (Lamentowicz et al., 2016), where we tested how microbial communities respond to experimentally manipulated water table depth.

**Table 1:** Moisture content differences between plots in Forbonnet and Linje. Sphagnum moisture content (%) was expressed as the difference between the fresh (FM) and the dry mass (DM) relative to the fresh mass: (FW−DW)/FW

Segment	Site	Hydrology	Mean	Median	Max	Min	*SE*
Upper *Sphagnum* segment	Forbonnet	MW	85.08	85.45	91.60	78.00	2.45
		MD	77.28	77.05	93.10	59.60	5.61
	Linje	W	92.13	89.90	96.60	89.90	1.41
		NAT	63.57	62.00	71.40	62.00	1.57
		ED	10.60	NA	NA	NA	NA
Lower *Sphagnum* segment	Forbonnet	MW	89.35	90.60	92.10	81.80	1.54
		MD	89.97	90.50	92.00	87.70	0.72
	Linje	W	91.10	91.10	91.10	91.10	0.00
		NAT	83.90	NA	NA	NA	NA
		ED	68.10	NA	NA	NA	NA

In Forbonnet, we used two different microhabitats, *that is,* hummocks and lawns, respectively, to compare naturally wet‐adapted communities to dry‐adapted ones. Lawns were flat and dominated by a homogeneous *Sphagnum fallax* carpet with a low abundance of vascular plants (i.e., *Eriophorum vaginatum, Andromeda polifolia, Vaccinium oxycoccos, and Scheuchzeria palustris*). Hummocks were bumpy and also dominated by a homogeneous *S. fallax* carpet, although small patches *of S. magellanicum* could be found. *A. polifolia, V. oxycoccos*,* Calluna vulgaris,* and *E. vaginatum* were the dominant vascular plant species in hummocks (Buttler et al., 2015). As a result of these micro‐topographical patterns, the water level was on average three centimeters lower in hummocks than in lawns (Buttler et al., 2015; Delarue et al., 2015); equivalent to a 9% reduction in *Sphagnum* moisture (see below for details) content in the top three centimeters (Table 1). In total, 12 plots (1 m^2^ each) were randomly assigned to these two microhabitats, six in hummocks (hereafter named “moderately dry, MD”) and six in lawns (hereafter named “moderately wet, MW”).

Linje peatland was dominated by a homogeneous *S. fallax* carpet with a vascular plant layer dominated by *E. vaginatum, V. oxycoccos,* and *A. polifolia*. We selected 18 plots (1 m^2^ each) within the bog with homogeneous plant species assemblage and micro‐topography. The water‐table manipulation consisted of three treatments randomly assigned to these plots, each with six replicates: wet (hereafter “W”), natural conditions (hereafter “NAT”), and extremely dry (hereafter “ED”) (Figure S2). In each of the manipulated plots (W, NAT, ED), we cut and removed four peat blocks of 50 × 50 × 30 cm. In the wet plots, we then excavated further 10 cm of peat and replaced the peat blocks in their initial location but in a 10‐cm lower position. The excavated peat from the W plots was then added to the ED plots following the same procedure to have the peat blocks lying 10 cm higher. In NAT plots, the four peat blocks were cut, removed, and replaced immediately in the same position. Thus, all plots were exposed to the same cutting and moving disturbance. All plots were bordered with a 15‐cm‐high plastic sheet so as to maintain the structure of the moss carpet (Lamentowicz et al., 2016). As a result of the treatment, the *Sphagnum* moisture content in the upper segment in W was 45% higher than NAT while the moisture content in ED was 83% lower than NAT (Table 1). The experiment was set up in early May 2012. In addition to the manipulated plots, we selected six other plots that remained untouched (intact plots) to test for the peat cutting and moving effects on the biotic and abiotic variables that were measured. Overall, we did not find significant effects of the cutting and moving phases for most of the microbial variables we measured (Figures S3 and S4).

We sampled *Sphagnum* shoots on 26 June 2008 in Forbonnet and on 18 May 2012 in Linje (2 weeks after the experiment started), which corresponds to peak of microbial biomass in peatlands (Heal, 1964). In each plot, *ca*. 100 g fresh weight of *S. fallax* was sampled around 10 permanently marked spots (i.e., *ca*. 10 g per spot). This sampling design allowed us to obtain a composite sample, representative of the entire plot while minimizing the possible effect of spatial heterogeneity (Mitchell et al., 2000). In Linje, *S. fallax* was sampled at least 5 cm from the edge of each monolith to avoid any possible bias resulting from border effect. Each *Sphagnum* shoot was cut immediately after sampling into two segments: 0–3 cm (upper segment) and 3–6 cm (lower segment) from the capitula, fixed in 20 ml of glutaraldehyde (2% final concentration), and stored at 4°C in the dark. For enzyme activities, the samples were stored at 4°C directly after sampling and analyzed within a week. We quantified the *Sphagnum* moisture on 20 randomly picked *Sphagnum* shoots in each plot, weighted them fresh and dried after 48 hr at 80°C. Then, the *Sphagnum* moisture content (%) was expressed as the difference between the fresh (FM) and the dry mass (DM) relative to the fresh mass: (FW‐DW)/FW.

### 2.2 Laboratory analyses

Microbial consumers (testate amoebae, ciliates, rotifers, and nematodes), bacteria, and fungi were extracted following the method described in Jassey, Gilbert, Binet, Toussaint, and Chiapusio (2011). Briefly, each sample was shaken for 1 min, followed by pressing of the mosses and filtration using mesh (pores size: 250 μm). This first filtrate was kept in a capped tube. Subsequently 20 ml of glutaraldehyde (2%) was added to the mosses, shaken for 1 min, and filtered. The filtrate was left for 8 hr at 4°C to sediment, after which time the supernatant was added to the mosses while the sediment was added to the first filtrate. The process of shaking, filtration, and sedimentation was repeated six times, each time using the supernatant from the previous incubation. At each iteration, the sediment was added to the initial solution to obtain a final sample of 40 ml. The abundance of bacteria was analyzed using flow cytometry (bacterial counts) and epifluorescence microscopy (bacterial size). For the estimations, 10 ml sub‐samples were filtered using a mesh (pores size: 10 μm). For bacterial counts, sub‐samples were stained with SYBR Green (0.1 × final concentration) and incubated in the dark for 15 min. Sub‐samples (1 ml) were run at a speed of 2 μl/s at a count rate not exceeding 1,000 events/s. Epifluorescence microscopy was used to determine the size of bacteria: 1 ml sub‐samples were stained with DAPI (4,6‐ diamino‐2‐phenylindole; 3 μg/ml final concentration), incubated in the dark for 15 min, filtered on 0.2 μm black membrane filters, and examined by fluorescence microscopy at 1,000× magnification. Bacteria sizes were determined automatically using the ImageJ software and the plugin analyze particles (Rasband, 1997–2014). The abundance of microalgae, cyanobacteria, fungi and microbial consumers, as well as their identification to species level when possible, was carried out using a 3‐ml subsample and inverted microscopy (×400, Utermöhl method) with the appropriate taxonomic literature (Lynn, 2008; Mazei & Tsyganov, 2006; Radwan, Bielańska‐Grajner, & Ejsmont‐Karabin, 2004). For fungi, the number and length of hyphae and spores were quantified. We chose that approach because our aim was to estimate fungal biomass, which estimation using molecular methods is currently limited (Baldrian et al., 2013). The abundance of species/group was then converted into biovolume (μm^3^), calculated based on geometrical shapes using dimensions measured under the microscope (length or diameter; width, and height). Biovolumes were then converted to biomass (μg C) using conversion factors as given in Gilbert et al. (1998). The biomass data, obtained in micrograms of C per gram of *Sphagnum* dry mass (μg C/g DM), were then expressed into milligrams of C per square meter (mg C/m^2^) after quantifying the dry mass of *Sphagnum fallax* shoots per square meter.

### 2.3 Predator–prey mass ratio

The predator–prey mass ratio (PPMR) is an important parameter capturing the complex patterns of feeding links among species and individuals in a simplified way (Nakazawa, Ushio, & Kondoh, 2011). PPMR includes both the body size of individuals and their biomass and is therefore crucial for understanding the structure and dynamic of food webs (Barnes, Maxwell, Reuman, & Jennings, 2010; Brose et al., 2006). We used a low‐resolution PPMR based on the effect of predators on decomposers, calculated as follows:

$$\begin{array}{c} \text{PPMR}=\frac{\text{mean biomass of predators}}{\text{mean biomass of prey}} \end{array}$$

$$\begin{array}{cc} & \mathrm{predators}=\mathrm{testate}\mathrm{amoebae}+\mathrm{rotifers}+\mathrm{ciliates}+\mathrm{nematodes}\;\\ & \mathrm{prey}=\mathrm{fungi}+\mathrm{bacteria} \end{array}$$

We used ln(*y* + 1) transformed data to calculate this ratio. We chose this low taxonomic resolution because it only requires descriptive information about predator and prey masses, rather than individual‐level feeding information. Such information is indeed unknown for most microbial species in peatlands. In addition to its technical ease, the PPMR allows comparisons among sites and/or ecosystem types (Brose et al., 2006).

### 2.4 Phenoloxidase activity and total phenolic content

We used phenoloxidase activity as a proxy for microbial activity; we chose this enzyme activity in particular because it plays a major role in peatland C sequestration (Fenner & Freeman, 2011; Freeman et al., 2001). We estimated potential enzyme activities in upper and lower segments of *S. fallax* using a method developed by Criquet, Tagger, Vogt, Iacazio, and Le Petit (1999). Briefly, 2 g of *S. fallax* (fresh weight) was shaken in 50 ml of a solution of 0.1 mol/L CaCl_2_ with 0.05% of Tween‐80 and 20 g of polyvinylpolypyrrolidone for 1 hr. The suspension of each extract was centrifuged, and the supernatant was filtrated (0.2 μm) and concentrated in cellulose dialysis tubing (10 kDa molecular mass cutoff) covered with polyethylene glycol. Then, concentrated extracts were re‐suspended in phosphate buffer (pH 5.6) until 1/10 of the initial volume. Phenoloxidase activities were measured for 24 hr at 23°C by spectrophotometry using a 96‐well microplate. For each sample, eight replicate wells were included. For each sample, eight additional replicate wells containing 150 ml of boiled extract (3 hr at 90°C) were measured as control to account for the quenching effect. Each well contained 150 μl of enzyme (boiled) extract with 100 μl of L‐DOPA (10 mmol/L; ε^M^ = 37,000 M/cm; Jassey, Chiapusio, Gilbert, Toussaint, and Binet (2012)). The oxidation rate of L‐DOPA to quinone was monitored at 460 nm using a spectrophotometer. Potential phenoloxidase activities were expressed in nmol of substrate oxidized per hour, per dry mass of *Sphagnum* (nmolQ h^−1^ g^−1^ DM). Total phenolic content was quantified in living segments of *Sphagnum* (0–6 cm from the capitulum) following Jassey et al. (2011). Briefly, water‐soluble phenolic compounds were extracted from lyophilized *Sphagnum* using distilled water: 0.05 g dw in 10 ml shacked for 3 hr. We then quantified phenolic content with the Folin‐Ciocalteau reagent and expressed it in mg equivalent gallic acid (*A*_*760*_) per gram of *Sphagnum* dry mass (mg/g DM).

### 2.5 Numerical analyses

Prior all statistical analyses, a ln(*y* + 1) transformation of microbial biomass was applied (Jongman, ter Braak, & van Tongeren, 1995). The effects of water level treatments and sampling depth on each individual microbial group, phenoloxidase activity, and PPMR in both sites were tested with ANOVAs followed by post hoc analyses (Tukey's multiple comparisons of means test). Assumptions for normality and homoscedasticity of the data were previously tested.

The effects of water level treatments (*Sphagnum* moisture, arcsine transformed), depth, phenoloxidase, pH, and phenols on microbial assemblages (log‐transformed) in each site were assessed using redundancy analyses (RDA). The proportion of variance explained by explanatory variables was calculated using variance partitioning. The interactions between *Sphagnum* moisture and depth (*Sphagnum* segments) were also tested. The significance of the model and of each explanatory variable included in the model was tested using 999 permutations.

The response of individual microbial groups to shifts in moisture conditions in both peatlands (merged data sets) was assessed using linear regressions. Assumptions for normality and homoscedasticity of the data were previously tested.

We used the Indicator Value (IndVal) approach to quantify to what extent microbial groups are sensitive to changes in *Sphagnum* water content (i.e., wet or dry conditions; the two datasets were merged) (Dufrêne & Legendre, 1997). IndVal scores were calculated using the TITAN method (Baker & King, 2010) with permutation tests to assess the uncertainty in these scores. Then, the permuted IndVal scores were standardized (*z* scores) by subtracting the mean of randomized permutations from the observed IndVal, and dividing by its permuted *SD*. High *z* scores indicated high biomass of the microbial group when *Sphagnum* moisture is high, and low values indicate the opposite.

We used multiple (linear) regression to test linkages between phenoloxidase (dependent variable) and PPMR, pH, polyphenols (independent variables), which showed no significant effects of neither pH nor polyphenols. We made a new corrected model with phenoloxidase (dependent variable) and PPMR (independent variable).

All statistical and multivariate analyses were performed in RStudio (RStudio Team 2012) using R version 3.1.2 (R Core Team 2012) using vegan package (Oksanen et al., 2013).

## 3 RESULTS

### 3.1 Response of microbial communities to moisture gradient

**Figure 1:**
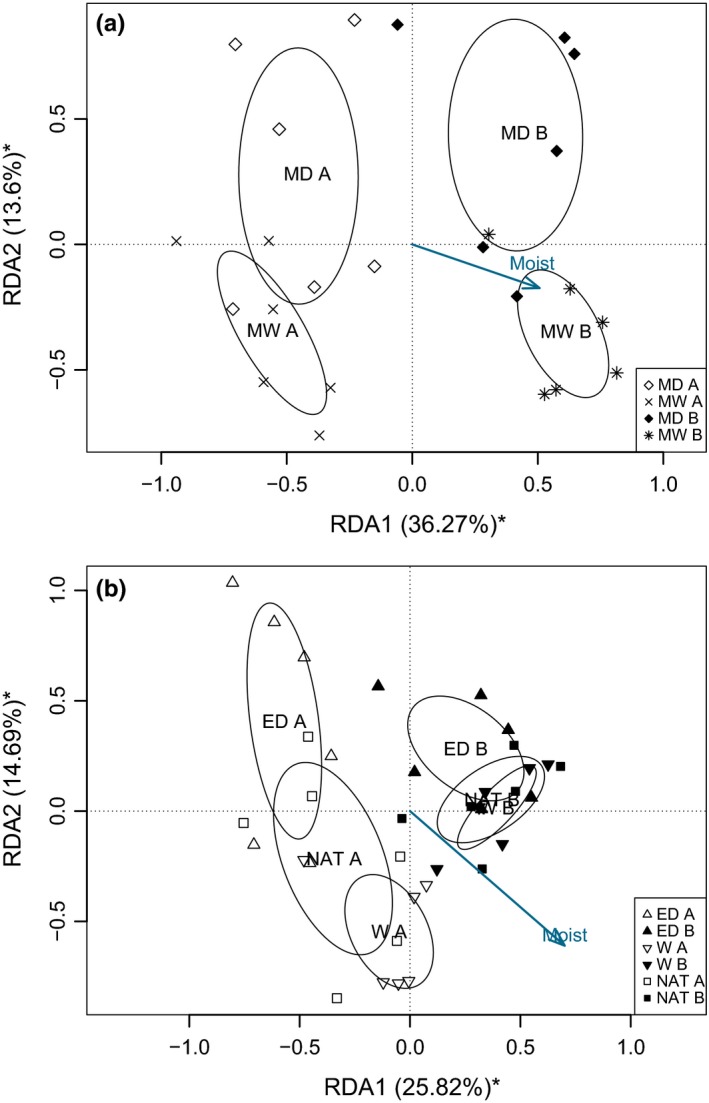
Redundancy analyses (RDA) of microbial communities (log‐transformed) in the observational study (Forbonnet) (a) and the extreme drought experiment (Linje) (b). Moist, *Sphagnum* moisture (%); MW, moderately wet plots (Forbonnet); MD, moderately dry (MD) (Forbonnet); W, wet (Linje); NAT, natural condition (Linje); ED, extreme drought (Linje); A, upper *Sphagnum* segments; B, lower *Sphagnum* segments. Ellipses represent standard errors of the (weighted) average of site scores around the centroid of each microhabitat or treatment × segments levels. In the brackets variation explained by each canonical axis is indicated and asterisks (*) indicate significance of the RDA axis

Microbial assemblages clearly differed among treatments and sampling depth in both sites (Figure 1, Table S1). In Forbonnet, the full RDA model significantly explained 41.9% of the variance in microbial community composition (*p = *.001) with depth (upper vs. lower segments) contributing most 32.4% (*p = *.001), while the fraction of variance explained by *Sphagnum* moisture content (8.7%; *p = *.002) and by phenoloxidase (9.1% ; p = .015) were lower. Phenols and pH were not significant in the model (*p = *.841 and *p = *.053). In Linje (Figure 1b), the full RDA model explained 39.7% of the variance (*p = *.001). *Sphagnum* moisture content explained 16.4% (*p = *.001) of the variance, depth (upper vs. lower segments) 20.6% (*p = *.001), and the fraction of variance explained by pH was 5.7% (*p = *.004; Figure 1b). Phenoloxidase and phenols were not significant in the model (*p = *.351 and *p = *.594).

**Figure 2:**
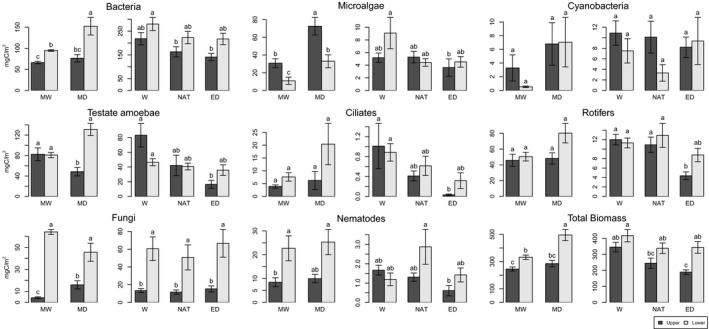
Biomass of microbial groups in the observational study and the experiment expressed in mg C/m^2^. MW, moderately wet plots; MD, moderately dry; W, wet (Linje); NAT, natural condition (Linje); ED, extreme drought (Linje). Letters indicate significant differences between treatments (*p < *.05, Tukey's test on ln(*y* + 1) transformed biomass data). Bars indicate standard errors

The patterns of microbial biomass differed significantly in relation to natural and experimental differences in moisture (Figure 2). Microbial consumers (testate amoebae, ciliates, rotifers, and nematodes) were particularly sensitive to soil moisture variations, especially in the upper *Sphagnum* segment. In the experimental site (Linje), the biomass of ciliates and rotifers significantly decreased from high to low moisture conditions in the upper segments (*p *<* *.05). By contrast, in the observational study (Forbonnet), the biomass of ciliates and rotifers tended to increase with decreasing moisture, particularly in lower segments; however, this difference was not significant *p* = .52 and *p* = .29, respectively; Figure 2). Testate amoeba biomass was positively correlated to moisture in both sites (MW 70% > MD; W 97% > NAT; ED < NAT 61%) in the upper *Sphagnum* segments (*p *<* *.05 in both sites). In the lower segment, the trend was the same in the experiment (W > NAT > ED) but was reversed in the observational study: 62% higher in MD as compared to MW (*p* > .05); these difference were, however, not significant. Nematode biomass did not differ significantly along the moisture gradient in either site.

In Forbonnet, bacterial biomass was 61% higher in the drier plots and this difference was significant in the lower *Sphagnum* segment (*p < *.05). In Linje, the opposite pattern was observed but differences were not significant (*p* = .15 and *p* = .63, upper and lower *Sphagnum* segment, respectively). Fungal biomass was higher in the low moisture plots in Forbonnet in the upper segments (*p < *.001) while the reverse trend was observed in the lower segment (nonsignificant). There was no clear pattern for fungal biomass in Linje. In the observational study, the biomass of microalgae was higher in the drier plots and the difference was significant in both, the upper and lower *Sphagnum* segments (*p < *.05). In Linje, the biomass of microalgae did not differ significantly along the moisture gradient in either *Sphagnum* segment. Cyanobacteria biomass did not differ significantly along the moisture gradient in either site.

**Figure 3:**
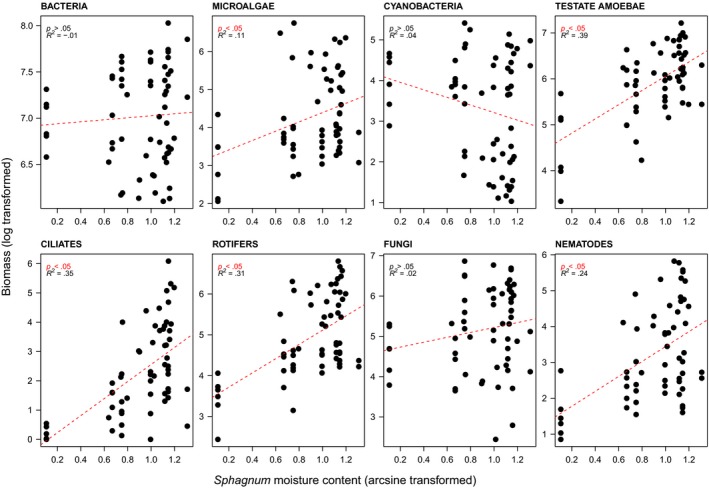
Linear regressions of the biomass of individual microbial groups to *Sphagnum* moisture conditions for the pooled data sets of Forbonnet and Linje peatlands

Most microbial groups were positively related to *Sphagnum* moisture (Figure 3). This response was, however, clearest for consumers such as testate amoebae, ciliates, rotifers, and nematodes (Figure 3). There was a rather large gap in the *Sphagnum* moisture gradient between ~20% and ~ 60%, which possibly biases our analysis due to a leverage effect. We thus tested the same relationships while excluding data points below 60% of *Sphagnum* moisture. The resulting analyses showed the same overall pattern (Figure S5) and did not either affect significantly the results of the RDAs (details not shown).

**Figure 4:**
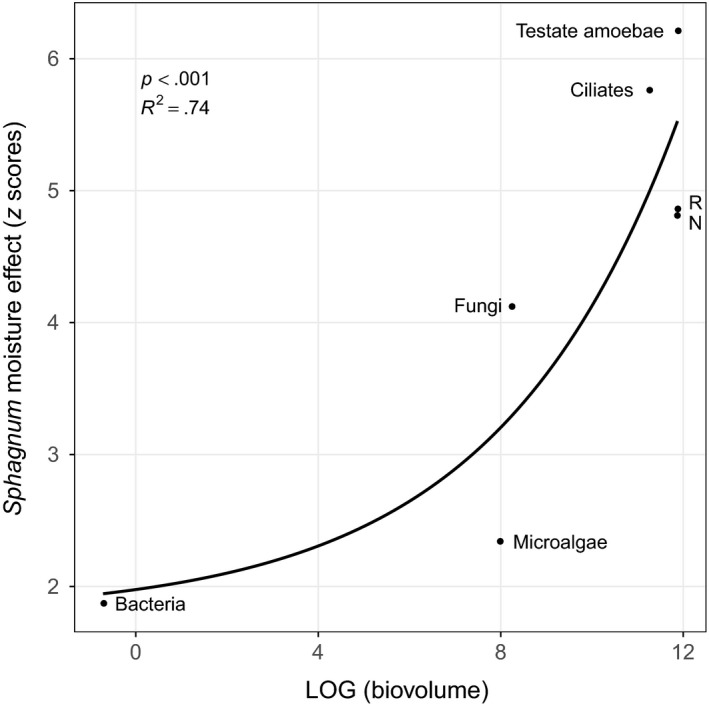
Relation between the response of each microbial group to shifts in *Sphagnum* moisture conditions (*z* scores) and their mean community size (mean community biovolume). IndVal scores were calculated using TITAN method (Baker & King, 2010) for each microbial group along the *Sphagnum* moisture gradient. Then, permuted IndVal scores were standardized as z scores. R, rotifers, N, nematodes

The sensitivity (z scores) of each microbial group to *Sphagnum* moisture was significantly correlated to their size (community averaged biovolume; Figure 4; *p < *.001, *R* ^2^ = .74). The most sensitive microbial groups were testate amoebae (*z*‐score = 6.21), ciliates (5.76), rotifers (4.86), and nematodes (4.81). The *z*‐scores of basal prey remained low for bacteria (1.87), fungi (4.12), microalgae (2.34), and cyanobacteria (1.51). The *z*‐score of cyanobacteria was not significant (*p *=* *.12), as such it is not depicted in Figure 4.

### 3.2 PPMR, phenoloxidase activity, and water‐soluble phenolic content

**Figure 5:**
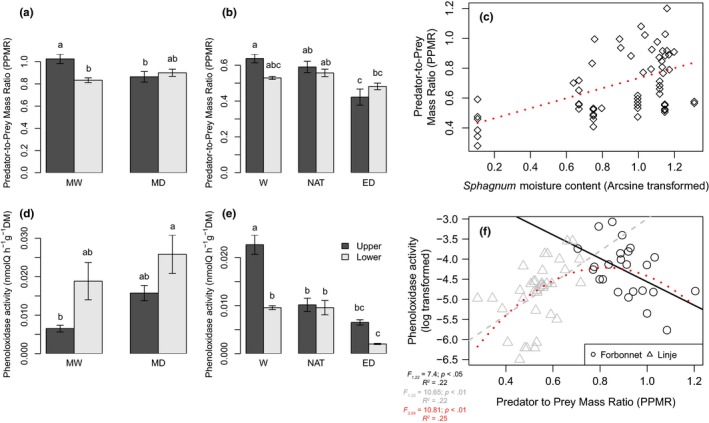
Response of phenoloxidase activity (a, b) and predator‐to‐prey mass ratio (PPMR) (d, e) to moisture in upper and lower *Sphagnum* segments in the two peatlands. MW, moderately wet plots; MD, moderately dry; W, wet (Linje); NAT, natural condition (Linje); ED, extreme drought (Linje). (c) Relationship between PPMR and *Sphagnum* moisture content (e) Relationship between PPMR and phenoloxidase activity (log‐transformed). Both upper and lower *Sphagnum* segments were combined. Triangles represent the drought experiment (Linje peatland) while circles the observational study (Forbonnet peatland). The red line in (f) represents the relationship between PPMR and phenoloxidases when both sites are combined

In Forbonnet, the PPMR decreased between MW and MD by 37% (*p < *.05) in upper segments but remained stable in lower segments (*p = *.60; Figure 5a). In Linje, similar tendencies were found with a decrease of PPMR in upper segments between W and ED treatments (−35%, *p < *.01) (Figure 5b). In lower segments, PPMR remained stable (Figure 5b), as in Forbonnet peatland. We further found significant relationship between PPMR and *Sphagnum* moisture content (*r *=* *.48, *p *<* *.001, Figure 5c) for the pooled data sets of Forbonnet and Linje peatlands.

The phenoloxidase activity in Forbonnet peatland was higher in the drier conditions than in the wetter ones, but this increase was stronger in upper segments (+60%, *p < *.01) than in lower segments (+30%, *p = *.89; Figure 5d). Water‐soluble phenolics showed similar patterns with a higher content in MD conditions (1.3 mg/g dw) compared to MW conditions (1.0 mg/g dw) (Figure S6). Extremely dry conditions had opposite effects on phenoloxidase activity in Linje. In upper segments, we found that phenoloxidase activity significantly decreased between W (0.023 nmolQ min^−1^ g^−1^ DM), NAT (0.01 nmolQ min^−1^ g^−1^ DM) and ED (0.006 nmolQ min^−1^ g^−1^ DM) treatments (Figure 5e). In lower segments, phenoloxidase activity strongly decreased between W/NAT and ED treatments (−97%, *p < *.01; Figure 5e). In the observational study, we further found an increase in phenoloxidase activity with depth, while in the experiment it was the opposite (Figure 5d,e). Water‐soluble phenolics did not vary among treatments with an average concentration of 2.0 mg/g dw (Figure S6).

Overall, phenoloxidase activity was not affected neither by pH nor polyphenols (*p = *.52 in both contrasts). We, however, found significant linkages between PPMR and phenoloxidase activity, but the direction of the relationship was opposite between both experiments (Figure 5f). While in Forbonnet, phenoloxidase activity was negatively related to PPMR (*r *=* *−.47; *p < *.05), a positive relationship was found in Linje (*r *=* *.47; *p < *.01). When results from the two sites were combined, a bell‐shaped relationship was observed (*R* ^2^ = .25; *p < *.01) showing that phenoloxidase activity remained low when PPMR was close to 0 or extremely high and was highest when PPMR was intermediate (Figure 5f).

## 4 DISCUSSION

Both naturally dry conditions and the sudden change in moisture regime triggered a set of significant belowground changes in both peatlands. On the whole, drought reduced the biomass of larger microbial consumers (testate amoebae), whereas that of bacteria and fungi tended to increase or remained stable. These changes modified the structure of the microbial food web with a decrease in the predator‐to‐prey mass ratio (PPMR) in both peatlands. We found that a decrease in PPMR can have divergent effects on microbial enzymatic activity and thus decomposition processes. In naturally dry conditions, a decreasing PPMR stimulated microbial activity, while in response to the extreme drought it reduced microbial activity. Our results showed that none of pH, polyphenols or *Sphagnum* moisture influenced phenoloxidase activity. Although we cannot exclude that other factors such as organic matter quality and nitrogen availability caused the differences in microbial enzyme activity (Sinsabaugh, 2010), PPMR remained the main driver of phenoloxidase activity in our study. In particular, our results suggest that microbial enzymatic activity resulting from food web structure is optimal only within a certain range of PPMR, and that different trophic mechanisms may be involved above and below this specific PPMR range. Further studies are necessary to disentangle possible mechanisms and estimate the optimal range of PPMR in which the phenoloxidase activity is the highest.

### 4.1 Community downsizing

We found contrasted microbial community structure along *Sphagnum* moisture content. In particular, the response of microbial communities to drought was mitigating with depth. Redundancy analyses clearly discriminated the microbial assemblages according to *Sphagnum* moisture content in surface *Sphagnum* while this was less pronounced in the lower *Sphagnum* segments, especially in the drought experiment. This indicates that the response of microbial communities to drought might be dependent on differences in total moisture, oxygen availability, or the frequency of water table fluctuations between the depths. Indeed, the upper section of the moss carpet often experience strong fluctuations in moisture content while moisture variability in the lower *Sphagnum* segments is less important (Mitchell & Gilbert, 2004). During summer (dry) period, lower amount of precipitation combined with a lower water table depth represents a physical limitation for *Sphagnum* water capillarity (Hájek, 2014). Microbial assemblages in the top surface then must face longer period of drought than those living deeper. These findings further suggest that the surface microbial communities have a great potential to monitor changes in peatland functioning due to drought or direct impacts such as drainage and the effect of restoration efforts (Geisen et al., 2017).

According to our expectations, the response of microbial assemblages to drought differed in relation to their trophic level. In particular, the larger the microbial consumers, the more they were sensitive to water deficit at the surface of *Sphagnum* peatlands. This phenomenon, known as “community downsizing” (Lindo, 2015; Sheridan & Bickford, 2011), was reported as a universal response to global warming (Gardner, Peters, Kearney, Joseph, & Heinsohn, 2011). Indeed, warming‐induced declines in mean body size members within a given community have been reported in numerous ecosystems (Gardner et al., 2011; Yvon‐Durocher et al., 2011), including peatlands (Jassey et al., 2013; Lindo, 2015; Mulot et al., 2017). While community downsizing under warming is mostly determined by changes in individual growth rates, as well as competitive and predatory interactions within the food web (Ohlberger, 2013), it likely results from physical changes (e.g., habitat loss) under drought (Schimel et al., 2007). A decrease in the water film thickness around *Sphagnum* leaves potentially reduces both the living and hunting spaces of microbes, thereby impacting preferentially larger individuals. These results are further supported by previous findings on testate amoebae where similar relationships between water table depth and the size of organisms were observed on short (Bonnet, 1973) and very long term (Fournier, Lara, Jassey, & Mitchell, 2015).

The loss of larger microbial consumers reduced predator–prey mass ratios. This suggests a destabilization of the peatland food web structure and functioning through trophic cascades, as previously shown in aquatic environments (Woodward, Papantoniou, Edwards, & Lauridsen, 2008), soil (Sauvadet et al., 2016), and boreal forests (Kardol, Spitzer, Gundale, Nilsson, & Wardle, 2016). Particularly, we found that drought decreased predator–prey mass ratios in both peatlands but as a result of differential responses of predators. The loss of larger microbial consumers (mostly testate amoebae) under extreme drought resulted in secondary extinction cascades in ciliates and rotifers. This suggests that extreme drought may induce trophic cascades where the loss of larger species had negative effects on the trophic levels below while their food resources are still high and top‐down regulation lower (Riede et al., 2011). Another explanation might be drought induced subtler indirect effects that do not involve species’ extinctions and rather modify species’ physiology which may cause changes in allocation and fate of C (Schimel et al., 2007). On the opposite, the loss of larger consumers in the naturally dry conditions had positive effects on the trophic levels below with the increase in small bacterial feeders (ciliates). Hence, novel trophic and competing interactions emerged along with community downsizing, which ultimately may alter community dynamics and functions (Lurgi, López, & Montoya, 2012; Sauvadet et al., 2016).

### 4.2 Drought and peatland functioning

Contrasting effects of drought on phenoloxidases have been found so far (Freeman et al., 2001; Wang, Richardson, & Ho, 2015), including this study. We found that moderate dry conditions had positive effects on phenoloxidase activity while extremely dry conditions had opposite effects. Previous studies showed that beneficial effects from drought on phenoloxidases such as increasing oxygen availability and pH, and shifts in dissolved organic matter quality (Dieleman, Branfireun, McLaughlin, & Lindo, 2016; Fenner & Freeman, 2011) can be offset by a loss of connection between the enzymes and their organic substrates when organic particles are too dry (Toberman et al., 2008). The latter effect might explain why phenoloxidase activity dropped under extremely dry conditions although we did not find a significant effect of *Sphagnum* moisture on enzymatic activity. An alternative or additional cause for the reduced phenoloxidase activity under extreme drought could be imputed to altered soil food web structure. Our findings evidence unrecognized food web mechanisms that may regulate enzymeatic activities. We demonstrated that phenoloxidase activity was highest under a specific range of PPMR, suggesting that bacterial and fungal enzymatic efficiency was dependent on a balance in microbial stocks. This further suggests that specific trophic mechanisms may influence soil enzyme activity. Particularly, drought‐promoted phenoloxidase activity when drought‐induced community downsizing was concomitant with the increase in smaller bacterial feeders. Hence, intensified bacterial grazing by ciliates may have enhanced bacterial turnover, and subsequently, enzymatic secretions involved in the decomposition of organic matter such as phenoloxidases. Indeed, shifts in the size structure of communities toward populations of smaller individuals has been shown to induce trophic cascades that increase trophic interactions and modify biomass distribution across trophic levels (Jochum, Schneider, Crowe, Brose, & O'Gorman, 2012), further stimulating bacterial and fungal enzymatic secretions (Trap et al., 2016). Similarly, other studies reported that a decrease in larger peatland microbial consumers but not of smaller ones was linked to higher concentrations of dissolved organic carbon and nutrients in the pore water and interpreted such results as an indication of the importance of food web size structure in driving microbial turnover, and, thus enzymatic secretions (Lamentowicz, Bragazza, Buttler, Jassey, & Mitchell, 2013). One explanation is that secondary extinction cascades potentially decreased bacterial and fungal enzymatic excretions because of a weaker top‐down stimulation effects on decomposers. From these results, we conclude that drought enhances microbial activity due to a decrease in the predator–prey mass ratio up to a breaking point when it reduces microbial activity. The loss of larger microbial consumers in response to moderate and the extreme droughts had similar implications for the community size structure, but this ultimately likely affected the mechanisms behind community dynamics and functions.

### 4.3 Conclusions

Peatlands accumulate carbon primarily due low decomposition rates (Davidson & Janssens, 2006). Studying the distribution patterns of the microscopic organisms responsible for this function, and their responses to experimental moisture manipulation and natural moisture gradients bring us closer to understanding the mechanisms by which climate warming is modifying the functioning of peatlands. Primary decomposition is mediated mainly by fungi and bacteria (Bardgett & Wardle, 2010). Regarding bacteria and fungi as only decomposers is obviously a simplification of the biotic interactions that drive key ecosystem processes such as decomposition. Sound estimates of the relative biomass of different microbial groups, which, as we show is essential to understand ecosystem functioning, are best achieved using direct microscopic observations. Evidences arise suggesting that microbial consumers in soils play a key role in regulating ecosystem processes such as nitrogen and carbon cycling (Geisen, 2016; Geisen et al., 2017), and that their role was oversimplified (Geisen, 2016). Our research emphasizes the underestimated role of the predator‐to‐prey mass ratio under changing climate.

## CONFLICT OF INTEREST

None declared.

## AUTHORS’ CONTRIBUTIONS

M.L., A.B., EADM, B.C., S.S., M.S., V.E.J.J and D.G. conceived the ideas and designed the methodology; M.K.R. and V.E.J.J. collected the data with M.L., D.G., G.C, P.B. and M.M. support; M.K.R. and V.E.J.J. analyzed the data; M.K.R. led the writing of the manuscript with substantial contribution from V.E.J.J.; all authors commented the manuscript. V.E.J.J; M.L., E.A.D.M., and A.B. revised it critically for important intellectual content.
