# Supplementary figures and images for: Predator–prey mass ratio drives microbial activity under dry conditions in Sphagnum peatlands
Source: Ecol Evol. 2018 May 12;8(11):5752–64. doi: 10.1002/ece3.4114 (PMC6010735; doi:10.1002/ece3.4114)

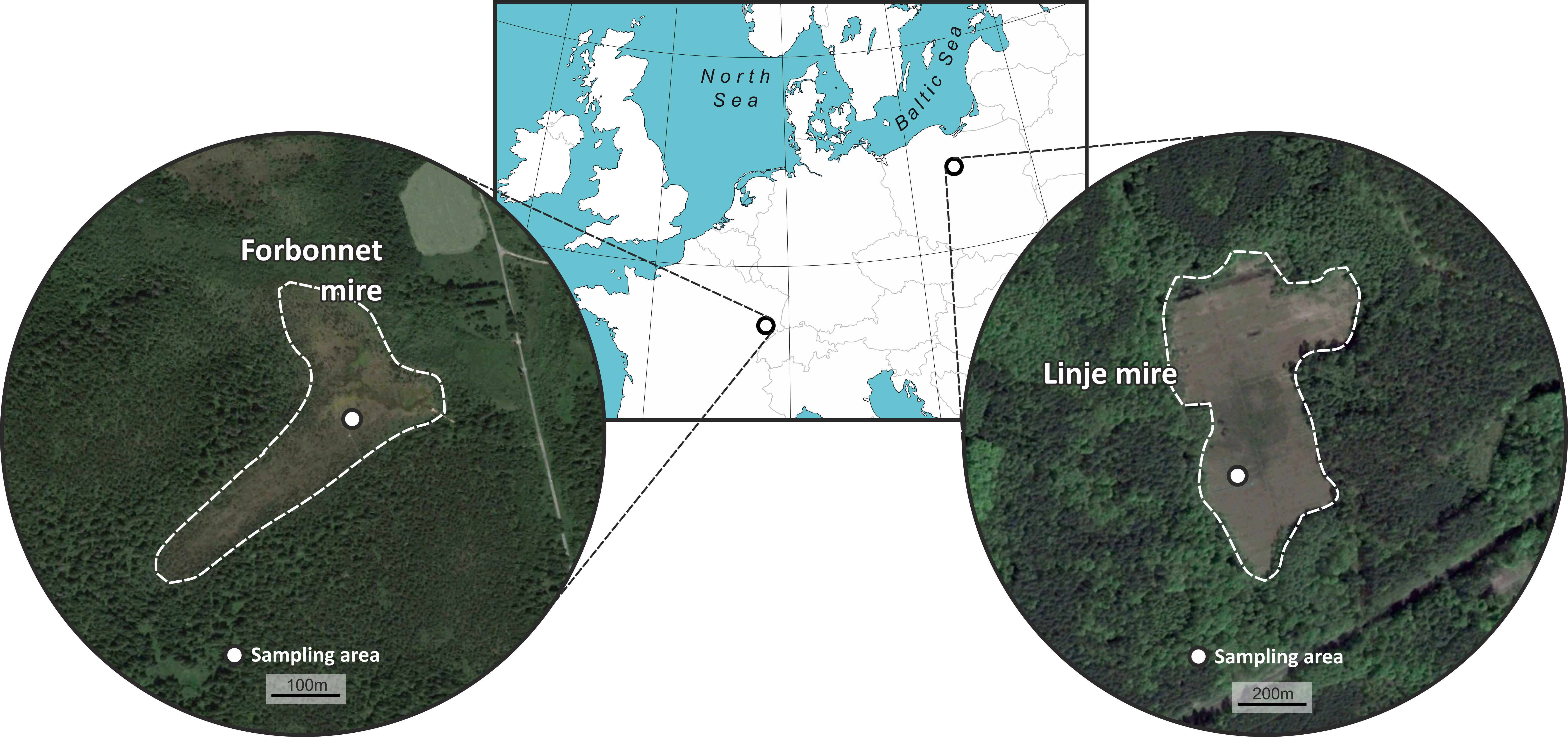

Supplement: Supplementary file 1 [file ECE3-8-5752-s001.jpg]

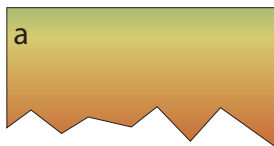

30 cm  
of peat

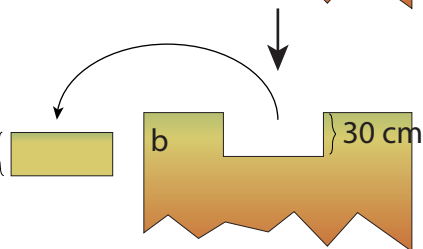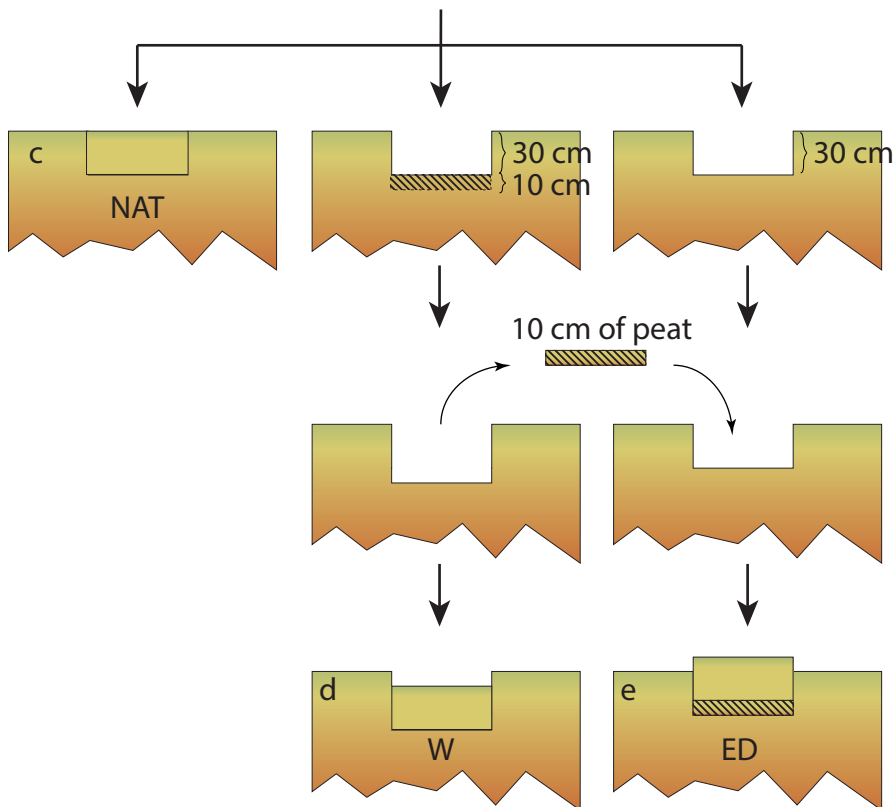

Supplement: Supplementary file 2 [file ECE3-8-5752-s002.pdf]

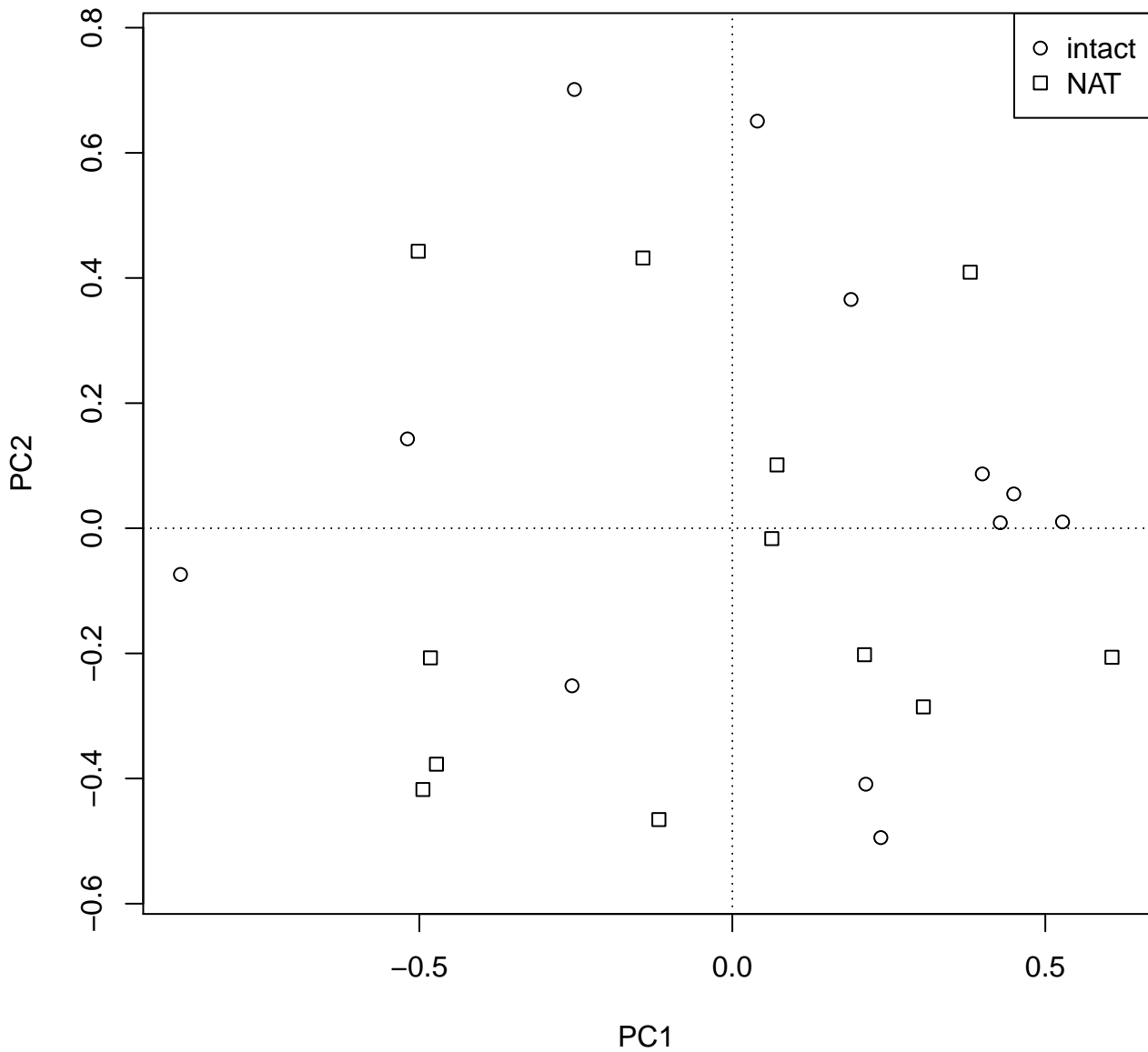

Supplement: Supplementary file 3 [file ECE3-8-5752-s003.pdf]

**Autotrophs**

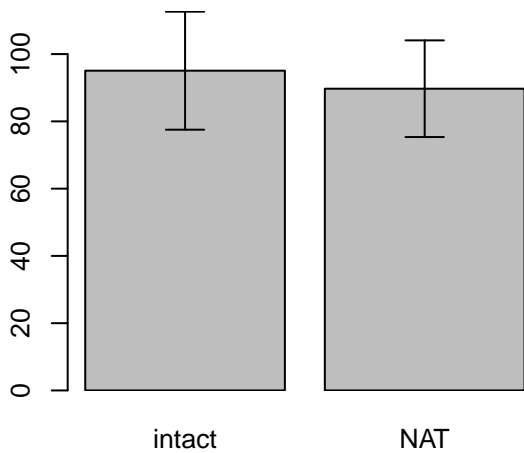

**Consumers**

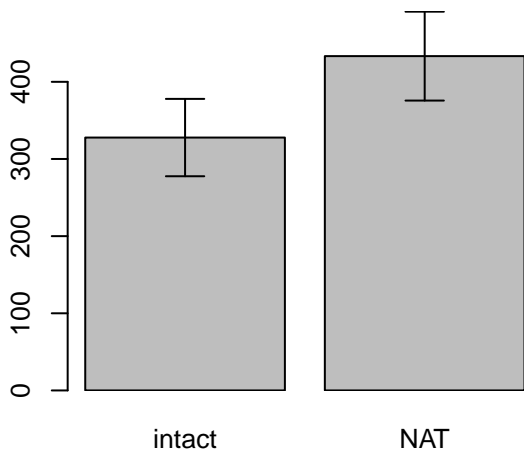

**Decomposers**

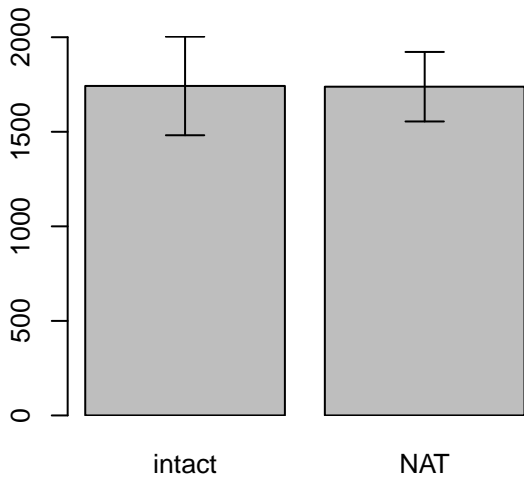

**Total\_biomass**

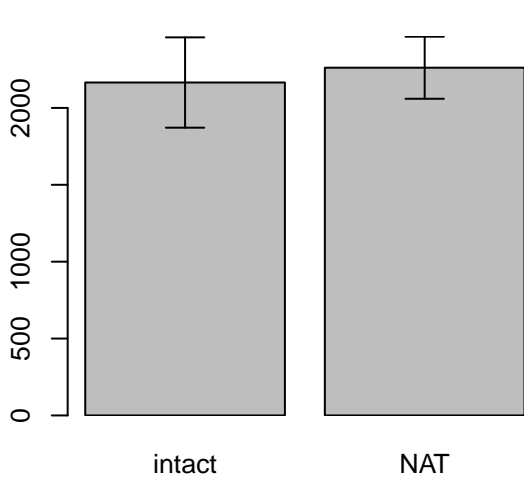

Supplement: Supplementary file 4 [file ECE3-8-5752-s004.pdf]

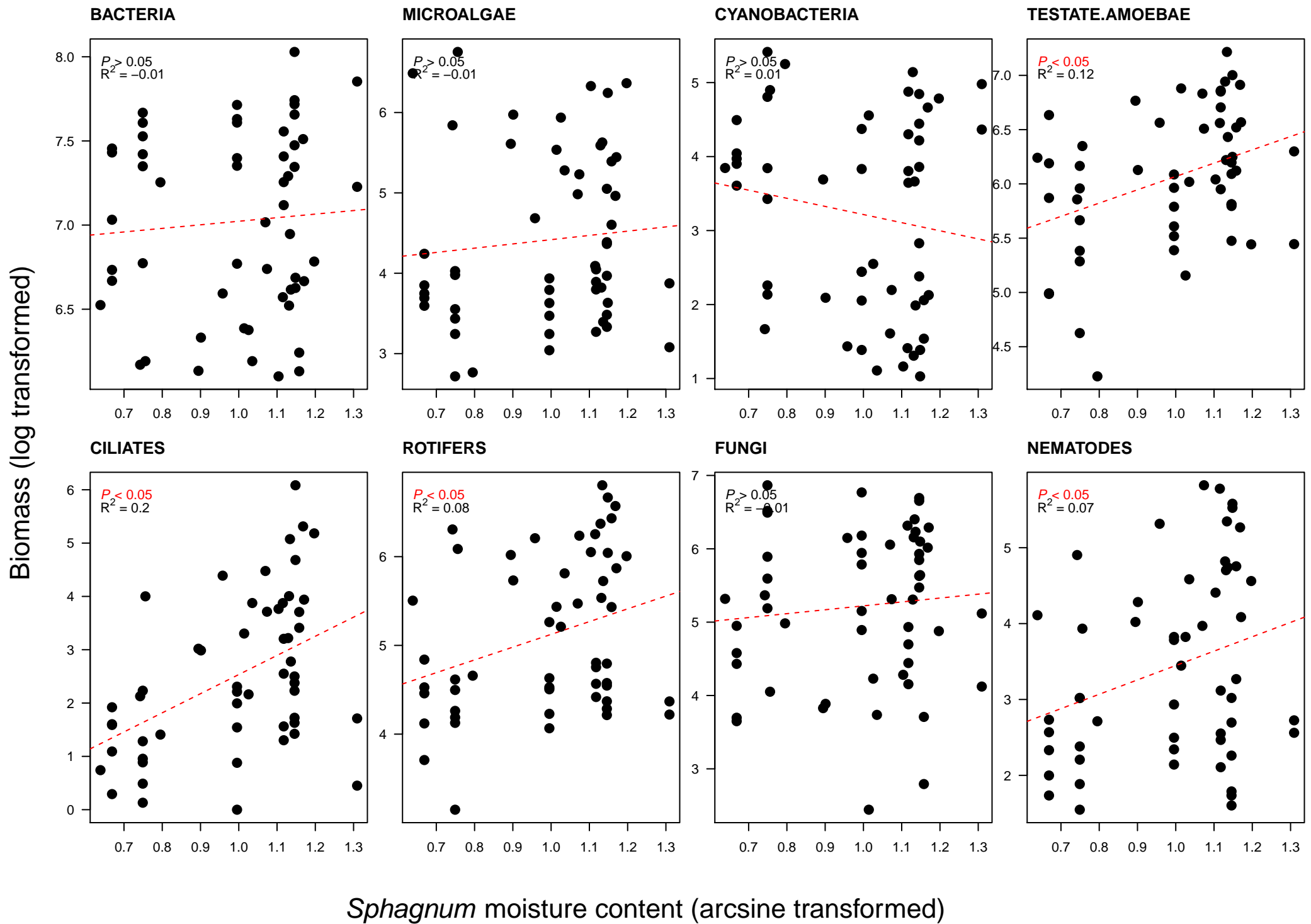

Supplement: Supplementary file 5 [file ECE3-8-5752-s005.pdf]

## Forbonnet

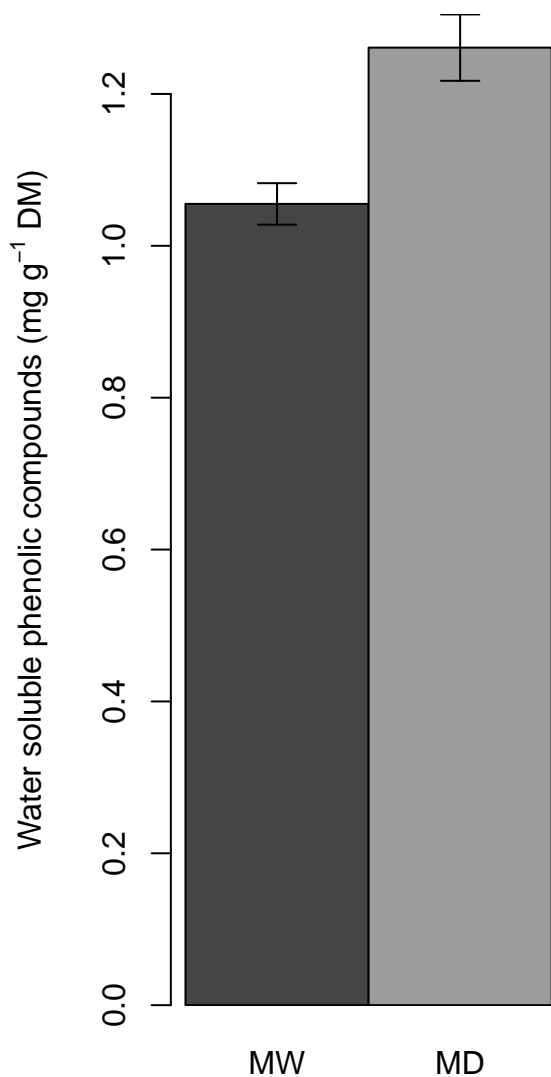

## Linje

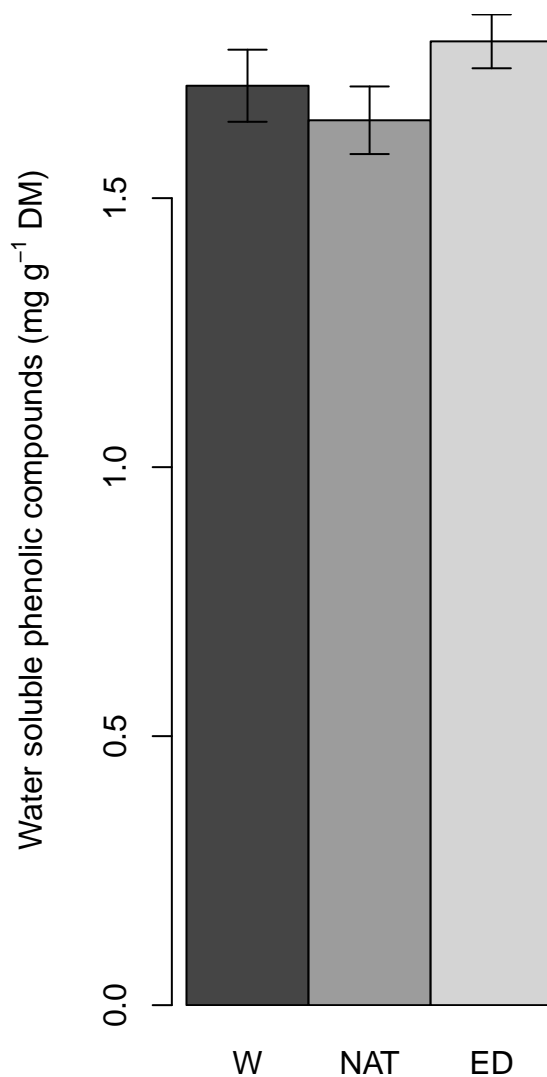

Supplement: Supplementary file 6 [file ECE3-8-5752-s006.pdf]
