# Supplementary material for: Predator–prey mass ratio drives microbial activity under dry conditions in Sphagnum peatlands
Source: Ecol Evol. 2018 May 12;8(11):5752–64. doi: 10.1002/ece3.4114 (PMC6010735; doi:10.1002/ece3.4114)
Supplement: Supplementary file 8 [file ECE3-8-5752-s008.docx]

| **Species** | **Forbonnet** | **Linje** |
| --- | --- | --- |
| *Amphitrema* *wrightianum* | + | - |
| *Arcella catinus* | - | + |
| *Arcella discoides* | - | + |
| *Arcella sp* | - | + |
| *Archerella flavum* | + | + |
| *Assulina muscorum* | + | + |
| *Assulina seminulum* | + | + |
| *Bullinularia indica* | - | + |
| *Centropyxis aerophila sphagnicola* | - | + |
| *Centropyxis sp* | - | + |
| *Corythion dubium* | - | + |
| *Cryptodifflugia crenulata* | - | + |
| *Cryptodifflugia oviformis* | - | + |
| *Cryptodifflugia sacculus* | + | - |
| *Difflugia globulus* | - | + |
| *Euglypha ciliata* | - | - |
| *Euglypha compressa* | + | + |
| *Euglypha rotunda* | - | + |
| *Euglypha sp* | - | + |
| *Euglypha strigosa* | + | + |
| *Heleopera rosea* | - | + |
| *Heleopera sphagni* | + | + |
| *Heleopera sylvatica* | - | + |
| *Hyalosphenia elegans* | + | + |
| *Hyalosphenia papilio* | + | + |
| *Microchlamys patella* | - | + |
| *Nebela collaris* | + | + |
| *Nebela militaris* | - | + |
| *Nebela penardiana* | + | - |
| *Nebela tincta* | + | + |
| *Nebela tincta major* | + | - |
| *Placocista spinosa* | - | + |
| *Physochila griseola* | + | + |
| *Pseudodifflugia gracilis* | + | - |
| *Trigonopyxis arcula* | - | + |
| *Trinema lineare* | - | + |
