# Supplementary material for: Predator–prey mass ratio drives microbial activity under dry conditions in Sphagnum peatlands
Source: Ecol Evol. 2018 May 12;8(11):5752–64. doi: 10.1002/ece3.4114 (PMC6010735; doi:10.1002/ece3.4114)
Supplement: Supplementary file 9 [file ECE3-8-5752-s009.docx]

## Supplementary material

Figure S1 Location of the studied peatlands: Forbonnet (France), Linje (Poland).

**Figure S2** Experimental water table manipulation in the Linje peatland: intact plot prior to the manipulation (a); cutting and removing peat blocks (b); natural condition (NAT) with peat blocks placed in the same position (c); wet treatment (W) with peat blocks lowered by 10 cm (d); extreme drought (‘ED’) with peat blocks uplifted by 10 cm (e).

**Figure S3** Principal Components Analysis (PCA) of microbial communities (log-transformed) in the NAT (natural condition; squares) and intact (control; circles) plots of Linje peatland. No differences are found between these two conditions in term of microbial community structure.

**Figure S4** Biomass of microbial groups in the NAT (natural condition) and intact (control) plots of Linje peatland. Microbial groups include: autotrophs (microalgae and cyanobacteria), consumers (testate amoebae, rotifers, nematodes and ciliates), decomposers (fungi and bacteria). There were no significant groups between intact and NAT plots for none of the groups considered (Anova tests, P > 0.05). Bars indicate standard errors.

Table S1 Biomass of different microbial groups (expressed in mgC · m^-2^; TA - testate amoebae), *Sphagnum* moisture content (Moisture) and phenoloxidase activity (PO). MW – moderately wet plots; MD – moderately dry; W – wet (Linje); NAT – natural condition (Linje); ED – extreme drought (Linje). Depth – *Sphagnum* segment: A – 0-3 cm (upper segment), B – 3-6 cm (lower segment).

**Figure S5** Linear regressions of the biomass of each individual microbial group to *Sphagnum* moisture conditions within the range 60-100% for the pooled data sets of Forbonnet and Linje peatlands.

**Figure S6** Water soluble phenolics content in Forbonnet and Linje peatlands.

Table S2 List of testate amoebae species found in Forbonnet and Linje peatland. + indicates that a given species was found in the peatland and - designates that it was not.
